# July Ladies’ Home Journal

**Published:** 1899-08

**Authors:** 


### *LITERARY NOTES.*

---

### JULY LADIES' HOME JOURNAL.

With its infinite variety of excellencies, the July *Ladies' Home Journal* appeals to every taste and touches upon every interest. It opens with "The Most Famous Little Town in America," which pictures many interesting spots in historic and literary Concord. There is a delightful view of social life in the Colonial days in "When Washington was Married," which brings to light many new, interesting facts. A series of almost incredible narratives in "The Moonlight King," tells of the follies and eccentricities of Ludwig II. of Bavaria. The gifts to our Government from foreign Powers are described in "Presents That Have Come to Uncle Sam." Ian Maclaren discusses the pulpit and the pew in an article on "How to Make the Most of Your Minister," and Katharine Roich writes of "The College-Bred Woman in Her Home."

The fiction of the July *Journal* includes a continuation of Anthony Hope's serial, "Captain Dieppe," the conclusion of "A College Courtship," the second of "Ol Peckham's Opinions," and a humorous portrayal of "The Valor of Brinley," by John Kendrick Bangs. "Entertaining in the Country," "How to be Pretty Though Plain," "What it Means to be a Dressmaker," "Birthday Parties," "A Boy's Club-House on the Water," are some of the seasonable, practical features. Mrs. S. T. Rorer writes on "Hasty Eating and Hurried Meals," and "Cooking Over All Sorts of Fuel," and Maria Parloa describes and pictures new and effective labor-saving devices for the home. "The Gossip of a New York Girl" details the very newest fancies in feminine attire, and "Pretty Stuffs for Midsummer Frocks" are described. Two pages are devoted to "Floral Porches and Vine-Clad Cottages," an attractive feature filled with suggestions for every homekeeper. By The Curtis Publishing Company, Philadelphia. Ten cents per copy; one dollar per year.

